# A bibliometric and visualized analysis of preoperative future liver remnant augmentation techniques from 1997 to 2022

**DOI:** 10.3389/fonc.2023.1185885

**Published:** 2023-06-02

**Authors:** Sihao Du, Zhenshun Wang, Dongdong Lin

**Affiliations:** Department of General Surgery, Xuanwu Hospital, Capital Medical University, Beijing, China

**Keywords:** bibliometric analysis, liver resection, future liver remnant (FLR), embolization, data mining

## Abstract

**Background:**

The size and function of the future liver remnant (FLR) is an essential consideration for both eligibility for treatment and postoperative prognosis when planning surgical hepatectomy. Over time, a variety of preoperative FLR augmentation techniques have been investigated, from the earliest portal vein embolization (PVE) to the more recent Associating liver partition and portal vein ligation for staged hepatectomy (ALPPS) and liver venous deprivation (LVD) procedures. Despite numerous publications on this topic, no bibliometric analysis has yet been conducted.

**Methods:**

Web of Science Core Collection (WoSCC) database was searched to identify studies related to preoperative FLR augmentation techniques published from 1997 to 2022. The analysis was performed using the CiteSpace [version 6.1.R6 (64-bit)] and VOSviewer [version 1.6.19].

**Results:**

A total of 973 academic studies were published by 4431 authors from 920 institutions in 51 countries/regions. The University of Zurich was the most published institution while Japan was the most productive country. Eduardo de Santibanes had the most published articles, and Masato Nagino was the most frequently co-cited author. The most frequently published journal was HPB, and the most cited journal was Ann Surg, with 8088 citations. The main aspects of preoperative FLR augmentation technique is to enhance surgical technology, expand clinical indications, prevent and treat postoperative complications, ensure long-term survival, and evaluate the growth rate of FLR. Recently, hot keywords in this field include ALPPS, LVD, and Hepatobiliary Scintigraphy.

**Conclusion:**

This bibliometric analysis provides a comprehensive overview of preoperative FLR augmentation techniques, offering valuable insights and ideas for scholars in this field.

## 1 Introduction

Insufficient future liver remnant (FLR) is a critical consideration that prevents patients from undergoing a major-hepatectomy. This is due to the increased risk of developing postoperative liver failure and mortality (1, 2). To overcome this limitation, regenerative liver surgery focuses on augmenting the FLR to an adequate size before performing the resection surgery. As far back as the 1980s, Kinoshita et al. (3) identified portal vein embolization (PVE) as a technique to safeguard the liver against tumor thrombi resulting from hepatocellular carcinoma (HCC). Makuuchi et al. (4) later suggested using PVE to enable surgical removal of primary liver tumors in patients with limited liver remnants. Since then, Preoperative PVE has been accepted as the standard approach for patients who are undergoing partial hepatectomy. However, there is a possibility of inadequate growth of FLR as well as tumor progression while waiting for the liver to regenerate, leading to the exploration of alternative techniques. Recently, newer strategies have emerged, with the most notable being associating liver partition and portal vein ligation for staged hepatectomy (ALPPS) and liver venous deprivation (LVD) (5–7).

Bibliometric analysis involves summarizing the data and publication features in a particular field, utilizing the available qualitative information (8, 9). This analysis enables researchers to comprehend the knowledge structure and pinpoint research fronts or hotspots in the area of study. Additionally, it assesses global scientific publications, thus exposing the latest advancements and trends over time within the field of study (10). To highlight its strengths and identify the knowledge domain and emerging trends in FLR augmentation, this study utilized the CiteSpace [version 6.1.R6 (64-bit)] and VOSviewer [version 1.6.19] tools. These tools helped us to create scientific knowledge-maps and analyze publications from 1997 to 2022.

## 2 Materials and methods

### 2.1 Data selection

To obtain pertinent articles, the Web of Science Core Collection (WoSCC) database was utilized due to its comprehensive bibliometric data and significant influence in this area of research (8–10). The search was conducted between 1 January 1997 and 31 December 2022, and had a limitation of article types: “Article” or “Review”, written in English. Boolean logic operators were employed to conduct the search with the following terms: (TS= (Hepatectomy) OR TS= (Hepatectomies)) AND (TS= (Portal vein embolization) OR TS= (PVE) OR TS= (Associating liver partition and portal vein ligation for staged hepatectomy) OR TS= (ALPPS) OR TS= (Liver venous deprivation) OR TS= (LVD) OR TS= (Radiation lobectomy) OR TS= (Transarterial embolizationportal vein embolization) OR TS= (TAE-PVE) OR TS= (FLR augmentation)). We selected the “Full Record and Cited References” format for the retrieved results and downloaded them in plain text document format. Then screen the retrieved papers again and exclude irrelevant literature. The summary of data source and selection was shown in **Table 1**. As the data was obtained directly from publicly available databases, no ethical approval was deemed necessary for this study.

**Table 1:** Summary of data source and selection.

Category	Specific Standard Requirements
Research database	Web of Science core collection
Citation indexes	SCI-EXPANDED
Searching period	January1997 to December 2022
Language	English
Searching keywords	(TS= (Hepatectomy) OR TS= (Hepatectomies)) AND (TS= (Portal vein embolization) OR TS= (PVE) OR TS= (Associating liver partition and portal vein ligation for staged hepatectomy) OR TS= (ALPPS) OR TS= (Liver venous deprivation) OR TS= (LVD) OR TS= (Radiation lobectomy) OR TS= (Transarterial embolizationportal vein embolization) OR TS= (TAE-PVE) OR TS= (FLR augmentation))
Subject categories	‘Surgery’ or ‘Gastroenterology Hepatology’ or ‘Oncology’
Document types	‘Articles’ and ‘Review Articles’
Data extraction	Export with full records and cited references in plain text format
Sample size (After Data Cleaning)	973

### 2.2 Data analysis and visualization

Currently, several bibliometric software are utilized, including CiteSpace, VOSviewer, UCINET and SciMAT (11). However, there is no agreement on which software is the most effective. To determine the most suitable software for our research requirements, we considered the unique features of each software and chose to employ CiteSpace [version 6.1.R6 (64-bit)] and VOSviewer [version 1.6.19] (12, 13) in our bibliometric analysis.

The VOSviewer software is a popular tool in bibliometric research because of its advanced visualization capabilities and data import/export functions from different sources to construct network-based maps (12). By studying citation links, bibliographic coupling, and co-occurrence, researchers can identify the clusters or themes present in the titles and abstracts of countries, institutions, and published papers (14). In this study, we utilized VOSviewer as our primary tool to visualize and analyze the key hotspots and evolution of nudge research. Network Visualization, Overlay Visualization, and Density Visualization were used for keyword co-occurrence and literature co-citations in the form of knowledge graphs.

In 2004, Prof. Chaomei Chen developed CiteSpace, a Java-based citation visualization software (15). This bibliometric analysis tool is capable of identifying potential research hotspots and trends in a specific field by generating a knowledge-map. The study examined various factors, such as the annual growth trend of publication outputs, institutions, countries/regions, journals, authors, occurrence of keywords, co-cited references, and reference bursts. To visualize the data, the researchers used CiteSpace [version 6.1.R6 (64-bit)].

## 3 Results

We gathered a sum of 973 publications, which consisted of 810 articles and 163 reviews. These investigations were published in 145 journals and were composed by 4431 authors who represented 920 organizations from 49 countries and regions. In total, these publications cited 12205 references from 1689 journals.

### 3.1 Growth trend of publications

**Figure 1:**
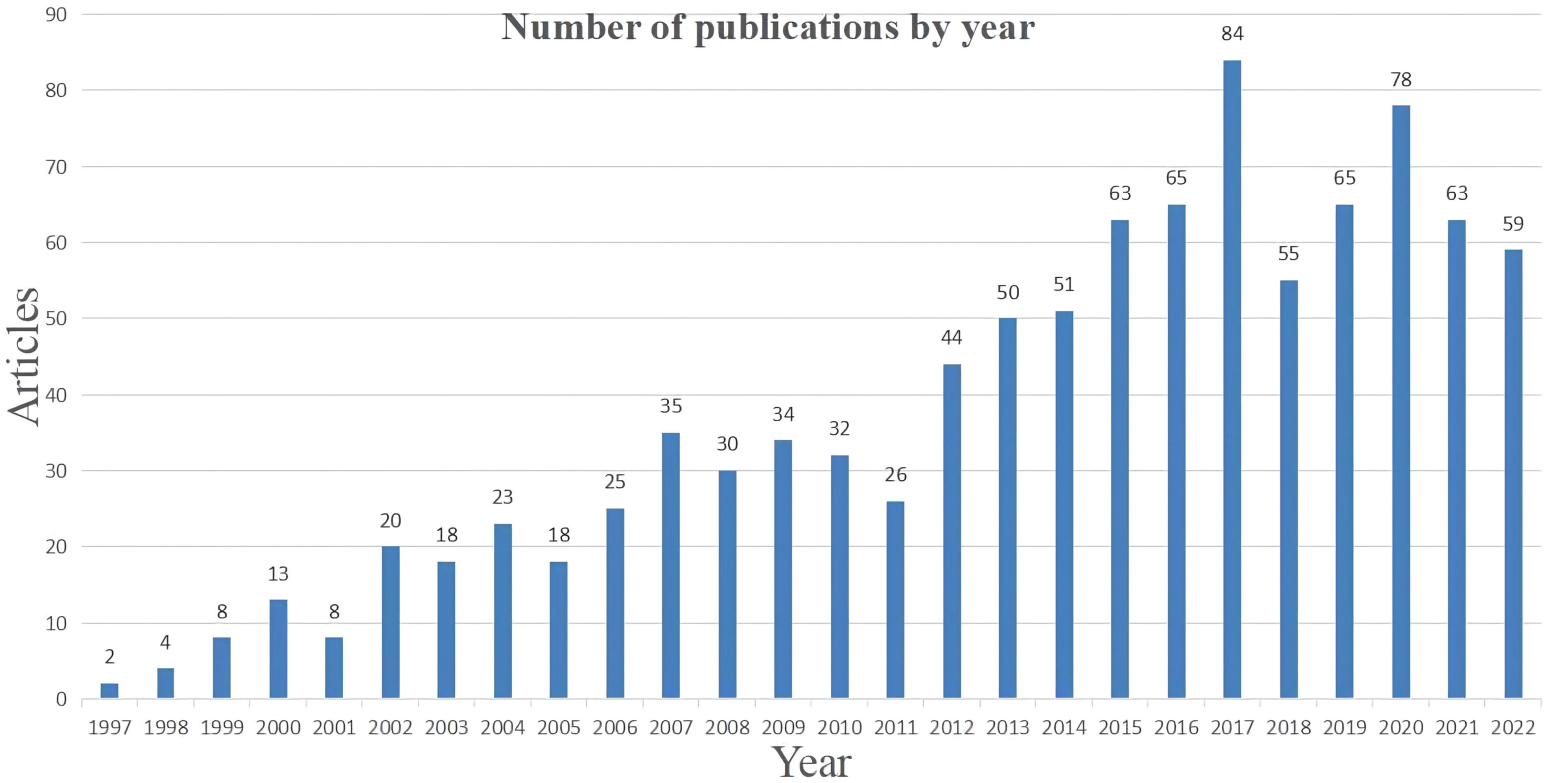
Number of publications by year.

According to **Figure 1**, the number of publications increased noticeably in 2012 and reached its peak in 2017, before experiencing a slight decline and stabilizing at around 60 papers per year. The increase in this trend may be associated with the advancement and utilization of ALPPS. The complete ALPPS procedure was initially reported by Schnitzbauer et al. in 2012, and further research has indicated that ALPPS generates a higher average FLR hypertrophy rate and significantly increased the probability of successful surgery compared to PVE (6, 16, 17). This finding sparked enthusiasm among researchers and eventually led to a peak of 84 publications in 2017. However, subsequent studies have demonstrated that ALPPS carries a significantly higher risk than PVE, with increased rates of associated complications and mortality (18, 19). This has prompted more rigorous selection criteria for patients deemed suitable for ALPPS, which may explain the reduction in the number of published literatures on the topic.

### 3.2 Top articles

The 10 most highly cited papers were captured based on their citations on Web of Science (**Table 2**). The number of citations varied and ranged from 846 to 343. One article was related to ALPPS while the remaining nine were related to PVE. However, the article about ALPPS had the highest total citations and average annual citations, indicating that it is a hot topic of recent research. The articles on PVE discussed its safety, effectiveness, application scenarios, long-term prognosis, and evaluation effects. These articles spanned from 2000 to 2008, suggesting that PVE was extensively discussed during this period. Over time, operational procedures improved and indications were better understood, eventually leading to a mature clinical application system.

**Table 2:** The 10 most cited publications.

Number	Author, year	Title	Techniques	Patients	Results	Conclusions	TC	TC per year
1	Schnitzbauer AA, 2012 (16)	Right portal vein ligation combined with *in situ* splitting induces rapid left lateral liver lobe hypertrophy enabling 2-staged extended right hepatic resection in small-for-size settings.	ALPPS	Colorectal liver metastasis: n=14 HCC: n=3 Cholangiocarcinoma: n=4 Gallbladder cancer: n=1 Other: n=3	Preoperative CT volumetry of the left lateral lobe showed 310 mL in median. After a median waiting period of 9 days, the volume of the left lateral lobe had increased to 536 mL, representing a median volume increase of 74%. The overall survival rate at 6 months after resection was 86%.	ALPPS is both safe and effective.	846	84.6
2	Farges O, 2003 (20)	Portal vein embolization before right hepatectomy - Prospective clinical trial	PVE	Colorectal liver metastasis: n=25 HCC: n=28 Cholangiocarcinoma: n=2	PVE in patients with chronic liver disease significantly decreased the incidence of postoperative complications (7 patients vs. 13 patients) as well as the intensive care unit stay (6 ± 3 vs. 15 ± 10 days, P < 0.05) and total hospital stay (13 ± 4 vs. 30 ± 15 days, P < 0.05) after right hepatectomy.	PVE is more suitable for patients with chronic liver disease.	522	27.47
3	Vauthey JN, 2000 (21)	Standardized measurement of the future liver remnant prior to extended liver resection: methodology and clinical associations.	PVE	Colorectal liver metastasis: n=14 HCC: n=3 Hilar cholangiocarcinoma: n=2 Gallbladder cancer: n=1	The future liver remnants increased after PVE (26% versus 36%, P < 0.01). Smaller size liver remnants were associated with an increase in postoperative liver function tests (P <.05) and longer lengths of hospital stay (P <.02).	CT scan with 3-dimensional reconstruction is a simple method of measurement provides an assessment of the liver remnant before resection.	486	22.09
4	Abulkhir A, 2008 (22)	Preoperative portal vein embolization for major liver resection: a meta-analysis.	PVE	Colorectal liver metastasis and other tumor: n=393 HCC: n=265 Cholangiocarcinoma: n=430	The overall morbidity rate for PVE was 2.2% without mortality. Four weeks following PVE, 85% patients underwent the planned hepatectomy.	PVE is a safe and effective procedure in inducing liver hypertrophy.	462	33
5	Azoulay D, 2000 (23)	Resection of nonresectable liver metastases from colorectal cancer after percutaneous portal vein embolization.	PVE	Colorectal liver metastasis: n=30	PVE was feasible in all 30 patients. There were no deaths. The complication rate was 3%. The post-PVE FLR was significantly increased compared with the pre-PVE value. Liver resection was performed after PVE in 19 patients (63%), with surgical death and complication rates of 4% and 7% respectively.	PVE is suitable for Colorectal liver metastasis.	410	18.63
6	Jaeck D, 2004 (24)	A two-stage hepatectomy procedure combined with portal vein embolization to achieve curative resection for initially unresectable multiple and bilobar colorectal liver metastases.	PVE	Colorectal liver metastasis: n=33	There was no operative mortality. Post-PVE morbidity was 18.1%; postoperative morbidity was 15.1% and 56.0% after first- and second-stage hepatectomy, respectively. The 1- and 3-year survival rates were 70.0% and 54.4%, respectively, in the 25 patients in whom the 2-stage hepatectomy procedure was completed.	2-stage hepatectomy procedure combined with PVE is suitable for patients with unresectable multiple and bilobar colorectal liver metastases.	394	21.8
7	Nagino M, 2006 (25)	Two hundred forty consecutive portal vein embolizations before extended hepatectomy for biliary cancer: surgical outcome and long-term follow-up.	PVE	Cholangiocarcinoma: n=150 Gallbladder cancer: n=90	There were no procedure-related complications requiring blood transfusion or interventions. 193 patients (132 cholangiocarcinomas and 61 gallbladder cancers) underwent hepatectomy. Seventeen (8.8%) patients died of postoperative complications. The 3- and 5-year survival after hepatectomy was 41.7% and 26.8% in cholangiocarcinoma and 25.3% and 17.1% in gallbladder cancer, respectively (P = 0.011)	PVE is suitable for patients with advanced biliary cancer.	381	23.8
8	Abdalla EK, 2006 (26)	Improving resectability of hepatic colorectal metastases: expert consensus statement.	PVE	/	Expert consensus statement of PVE	Expert consensus statement of PVE	379	23.6
9	Abdalla EK, 2002 (27)	Extended hepatectomy in patients with hepatobiliary malignancies with and without preoperative portal vein embolization.	PVE	Colorectal liver metastasis: n=23 HCC: n=5 Cholangiocarcinoma: n=8 Other: n=6	After PVE, FLR volumes increased significantly. There was no perioperative mortality and no statistical difference in the incidence of perioperative complications between those who did and those who did not undergo PVE (5 of 18 patients vs 5 of 24 patients). The overall 3-year survival was 65% and the median survival duration was equivalent in the 2 groups (40 vs 52 months for those who did vs those who did not undergo PVE).	PVE enables safe and potentially curative extended hepatectomy in patients who are marginal candidates for resection based on small liver remnant size.	348	17.4
10	Ribero D, 2007 (28)	Portal vein embolization before major hepatectomy and its effects on regeneration, resectability and outcome.	PVE	Colorectal liver metastasis: n=50 HCC: n=24 Hilar cholangiocarcinoma: n=14 Gallbladder cancer: n=6 Other: n=18	Major and liver-related complications, hepatic dysfunction or insufficiency, hospital stay and 90-day mortality rate were significantly greater in patients with a standardized FLR of 20 per cent or less or degree of hypertrophy of not more than 5 per cent compared with patients with higher values.	Degree of hypertrophy after PVE can help to assess the prognosis.	343	22.8

TC, Total citations; ALPPS, associating liver partition and portal vein ligation for staged hepatectomy; PVE, portal vein embolization.

### 3.3 Institutional and country contributions

**Figure 2:**
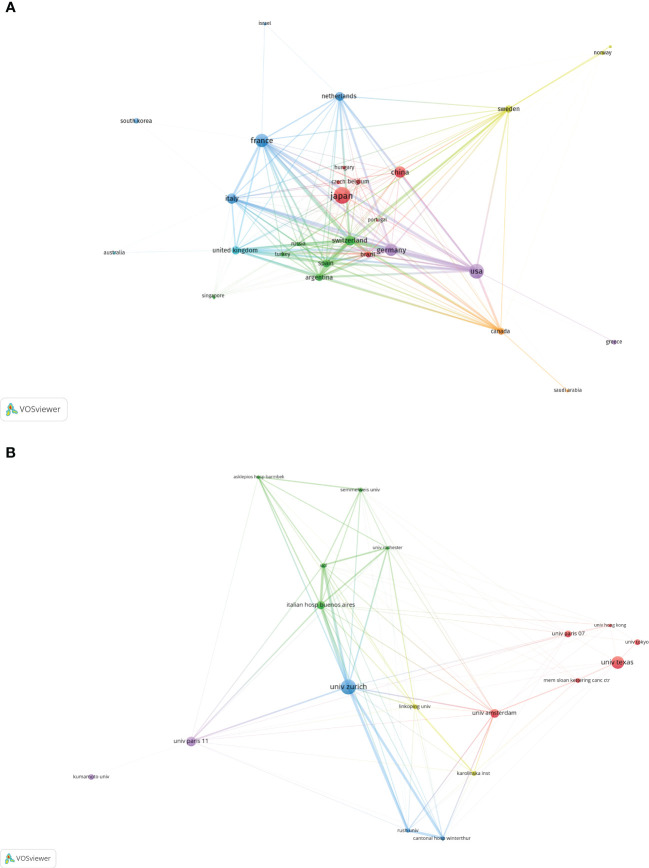
The cooperation relationships of Countries **(A)** and Institutions **(B)**. The size of the circular node in the graph corresponds to the number of publications it represents. The links between the nodes represent the degree of association, and thicker links indicate more frequent cooperation between two nations or organizations. The color of the node corresponds to various clusters.

The FLR augmentation technique has been explored by 49 different countries. The top 10 countries have been ranked based on their scientific impact, as **Table 3** indicates. Japan has the highest number of publications with 233 articles, followed by the United States of America (173 articles), France (142 articles), and Germany (122 articles). The USA publications were the most cited, with the French and Japanese papers close behind. **Figure 2** displays the collaboration network among countries, where the thickness of lines between the nodes indicates the level of cooperation. Although Japan occupies the central position in the network, the connecting lines to that node are all relatively thin, which indicates that Japan does not cooperate much with other countries. On the other hand, Switzerland holds the highest total link strength as indicated by the relatively thick connections to it, highlighting its central position in collaborating with other countries. Switzerland’s key partners include France, USA, Germany, Italy and Spain. Meanwhile, the USA and Germany exhibit a closely-coordinated collaboration, as can be seen in the **Figure 2A**.

**Table 3:** Top 10 countries with the most articles.

Rank	Country	Documents	Freq	Citations	Average Citation/Publication
1	Japan	233	23.9%	6573	28.21
2	USA	173	17.8%	8501	49.14
3	France	142	14.6%	7924	55.8
4	Germany	122	12.5%	4465	36.59
5	China	104	10.6%	1278	12.28
6	Italy	85	8.7%	2266	26.66
7	Switzerland	77	7.9%	2964	38.49
8	Netherlands	58	5.9%	1988	34.28
9	United kingdom	53	5.4%	2151	40.58
10	Spain	47	4.8%	1494	31.79

More than 920 institutions have applied FLR augmentation technique. A summary of the top 10 institutions, ranked by their contributions, is provided in **Table 4**. The University of Zurich, with 63 published articles, is the most productive academic institution, followed by the University of Texas (53 articles) and the University of Paris 11 (42 articles). The top institutions are fairly evenly distributed across continents, with Japan hosting all of the top Asian institutions. A network graph illustrating collaborative relationships between institutions is depicted in **Figure 2B**, with different colors representing those that have previously collaborated. University of Zurich occupies the central position in the network and has the highest centrality. The key partners of University of Zurich include University College London, Italian hospital Buenos Aires, Winterthur Cantonal Hospital and University of Rush.

**Table 4:** Top 10 institutions with the most articles.

Rank	Affiliations	Country	Documents	Citations	Average Citation/Publication
1	University of Zurich	Switzerland	63	2640	41.9
2	University of Texas	USA	53	4694	88.57
3	University of Paris 11	France	42	3739	89.02
4	University of Amsterdam	Netherlands	37	1117	30.19
5	Italian hospital Buenos Aires	Argentina	35	1734	49.54
6	University of Nagoya	Japan	34	1715	50.44
7	University of Paris 07	France	29	1703	58.72
8	University of Tokyo	Japan	28	1180	42.14
9	University of Kumamoto	Japan	24	457	19.04
10	University College London	United Kingdom	21	1047	49.86

### 3.4 Productive journals and co-cited journals

**Figure 3:**
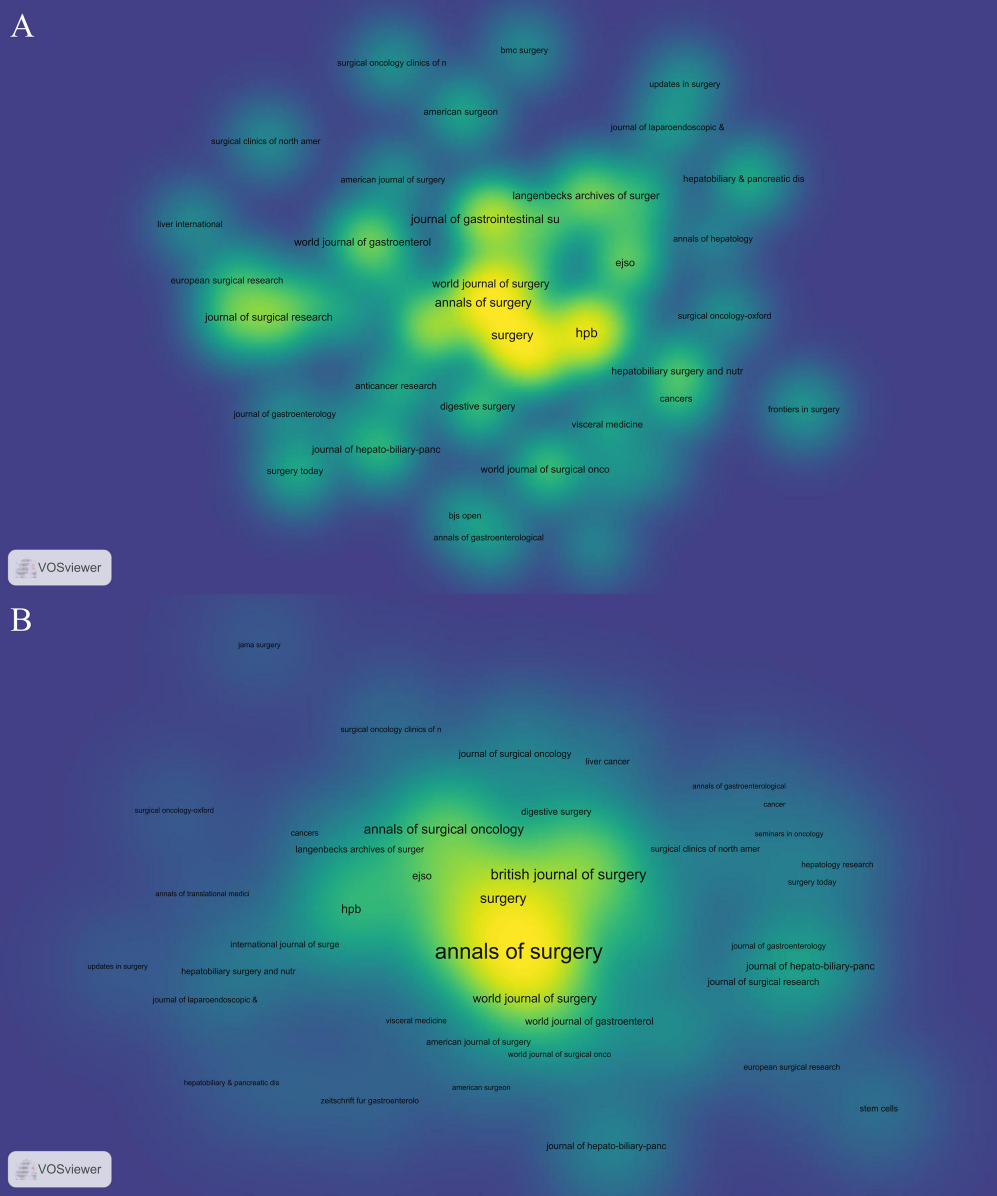
The density maps of most productive Journals **(A)** and Co-Cited Journals **(B)**. The darker and closer to yellow the node is, the more papers this journal has published or the more citations it has received.

We utilized VOSviewer [version 1.6.19] software to identify the academic journals with the highest number of published papers and co-citations in the field of FLR augmentation technique. Our statistical analysis revealed that there were 973 published documents across 145 different academic journals, as presented in **Table 5**, the top 10 journals and co-cited journals associated with this topic were identified, with HPB being the most frequently published journal with 65 citing articles, followed by Surgery (n = 59), Ann Surg (n = 58), Ann Surg Oncol (n = 46), and J Gastrointest Surg (n = 43). Additionally, there were five journals in the Q1 JCR division, with Ann Surg having the highest impact factor (IF = 13.787). We created a density map (**Figure 3A**) using the top 50 journals with the highest total link strength to effectively display the most productive journals. In **Table 6**, the journals with the highest frequency of citations were identified, with Ann Surg being the most cited journal with 8088 citations, followed by Br J Surg with 2693 citations, Surgery with 2448 citations, Ann Surg Oncol with 1959 citations, and J Gastrointest Surg with 1702 citations. Furthermore, eight journals were categorized in the Q1 JCR region, and Hepatology had the highest impact factor at 17.298. The density map shown in **Figure 3B** effectively illustrates the co-cited journals with the top 50 citations, as determined by the greatest total link strength.

**Table 5:** Top 10 journals with the most articles.

Rank	Journal	Documents	IF (2022)	JCR (2022)
1	HPB	65	3.842	Q1/Q3
2	Surgery	59	4.348	Q1
3	Ann Surg	58	13.787	Q1
4	Ann Surg Oncol	46	4.339	Q1
5	J Gastrointest Surg	43	3.267	Q2
6	Br J Surg	35	3.267	Q2
7	World J Surg	28	3.282	Q2
8	World J Gastroenterol	27	5.374	Q2
9	Langenbecks Arch Surg	26	2.895	Q2
10	J Surg Res	23	2.417	Q3

**Table 6:** The top 10 co-cited journals.

Rank	Co-cited journal	Citations	Documents	IF (2022)	JCR (2022)
1	Ann Surg	8088	58	13.787	Q1
2	Br J Surg	2693	35	11.122	Q1
3	Surgery	2448	59	4.348	Q1
4	Ann Surg Oncol	1959	46	4.339	Q1
5	J Gastrointest Surg	1702	43	3.267	Q2
6	HPB	1382	65	3.842	Q1/Q3
7	World J Surg	1317	28	3.282	Q2
8	J Am Coll Surgeons	1076	18	6.532	Q1
9	Hepatology	873	7	17.298	Q1
10	Eur J Surg Oncol	809	22	4.037	Q1

### 3.5 Productive authors and co-cited authors

**Figure 4:**
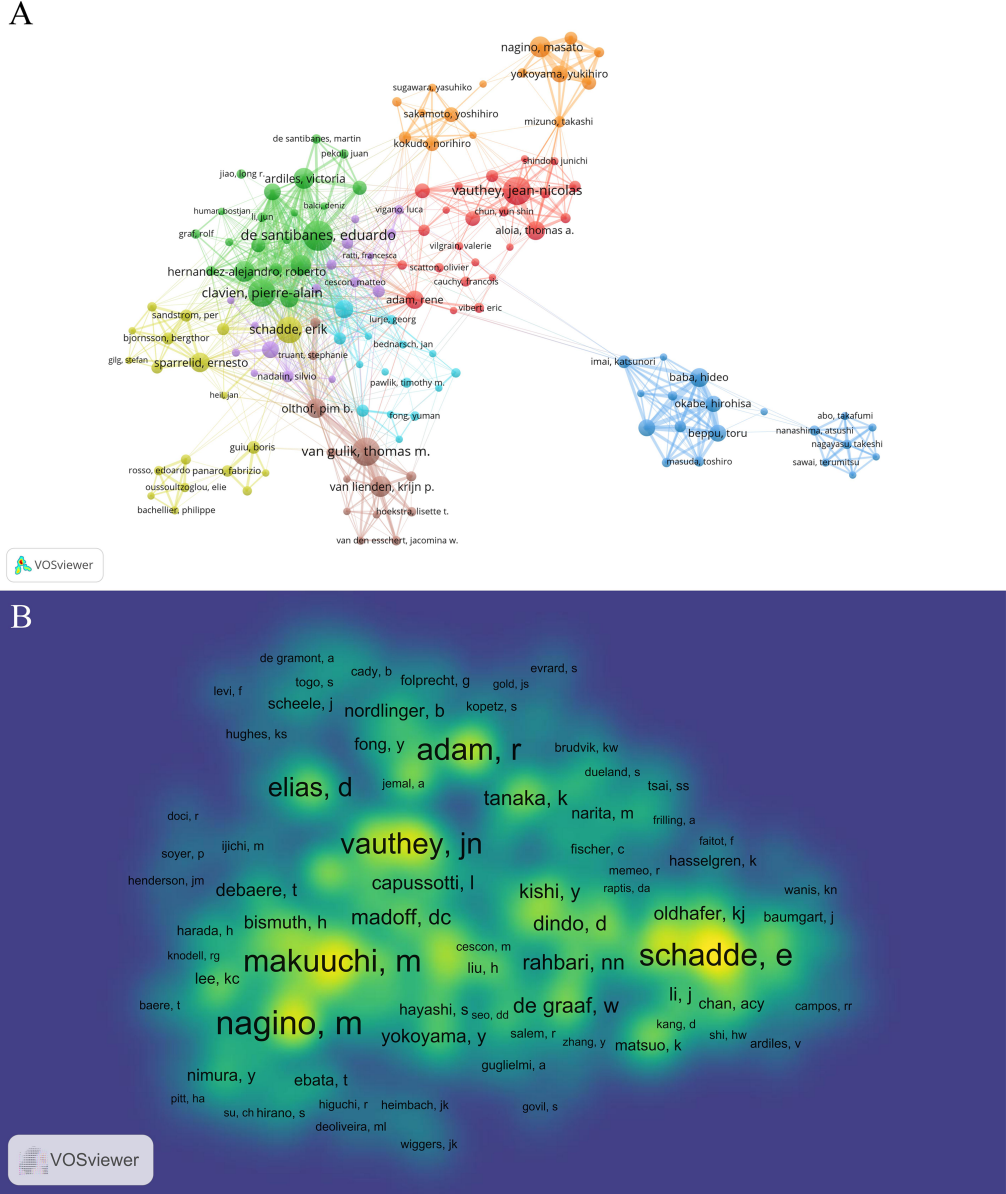
The co-occurrence maps in the FLR augmentation techniques. **(A)** Authors; **(B)** Co-authors. The size of the circular node in the graph corresponds to the number of publications it represents. The links between the nodes represent the degree of association, and thicker links indicate more frequent cooperation between two nations or organizations. The color of the node corresponds to various clusters.

Using VOSviewer [version 1.6.19], a bibliometric analysis was conducted to identify the most productive authors and co-cited authors of the FLR augmentation technique. The analysis retrieved 4431 authors, among whom Eduardo de Santibanes had the highest number of published papers (n=34), followed by Jean-Nicolas Vauthey (n=31), Thomas M van Gulik (n=31), Pierre-Alain Clavien (n=30), and Erik Schadde (n=28), as presented in **Table 7**. To draw the network map, authors who had a minimum of 5 documents were selected, resulting in 185 authors, as shown in **Figure 4A**. The map displayed different clusters with various colors, indicating the presence of close cooperation among authors. For instance, there was significant collaboration among clusters, such as Rene Adam and Katsunori Imai, Rene Adam and Thomas van Guilk, and Rene Adam and Eduardo de Santibanes. Moreover, active collaborations were observed among authors in the same cluster, including Jean-Nicolas Vauthey and Thomas Aloia, Masato Nagino and Yukihiro Yokayama, and Ernesto Sparrelid and Bergthor Bjornsson.

**Table 7:** The top 10 most prolific authors.

Rank	Author	Country	Documents
1	de Santibanes, Eduardo	Argentina	34
2	Vauthey, Jean-Nicolas	USA	31
3	Van Gulik, Thomas M.	Netherlands	31
4	Clavien, Pierre-Alain	Switzerland	30
5	Schadde, Erik	Switzerland	28
6	Hernandez-Alejandro,Roberto	USA	21
7	Ardiles, Victoria	Argentina	21
8	Nagino, Masato	Japan	21
9	van Lienden, Krijn P.	Netherlands	21
10	Linecker, Michael	Germany	20

Co-cited authors refer to two or more authors who were cited together in various studies. In this study, 7641 co-authors were identified, and the top 10 authors were all co-cited more than 200 times, as presented in **Table 8**. Masato Nagino had the highest number of co-citations (n=570), followed by Masatoshi Makuuchi (n=501), Erik Schadde (n=497), Rene Adam (n=486), and Jean-Nicolas Vauthey (n=429). To create a density map of the high-frequency co-cited authors, authors with at least 10 co-citations (n=584) were selected. The map revealed that the yellow regions of Masato Nagino, Masatoshi Makuuchi, and Erik Schadde were the darkest in **Figure 4B**, indicating that these authors were highly co-cited in this field.

**Table 8:** The top 10 co-cited authors.

Rank	Co-cited author	Country	Citations
1	Nagino, Masato	Japan	570
2	Makuuchi, Masatoshi	Japan	501
3	Schadde, Erik	Switzerland	497
4	Adam, Rene	France	486
5	Vauthey, Jean-Nicolas	USA	429
6	Abdalla, Eddie K	USA	363
7	Schnitzbauer,AA	Germany	357
8	Elias, D	France	335
9	Azoulay, Daniel	France	322
10	Shindoh, Junichi	Japan	299

### 3.6 Keyword co-occurrence, clusters, and evolution

**Figure 5:**
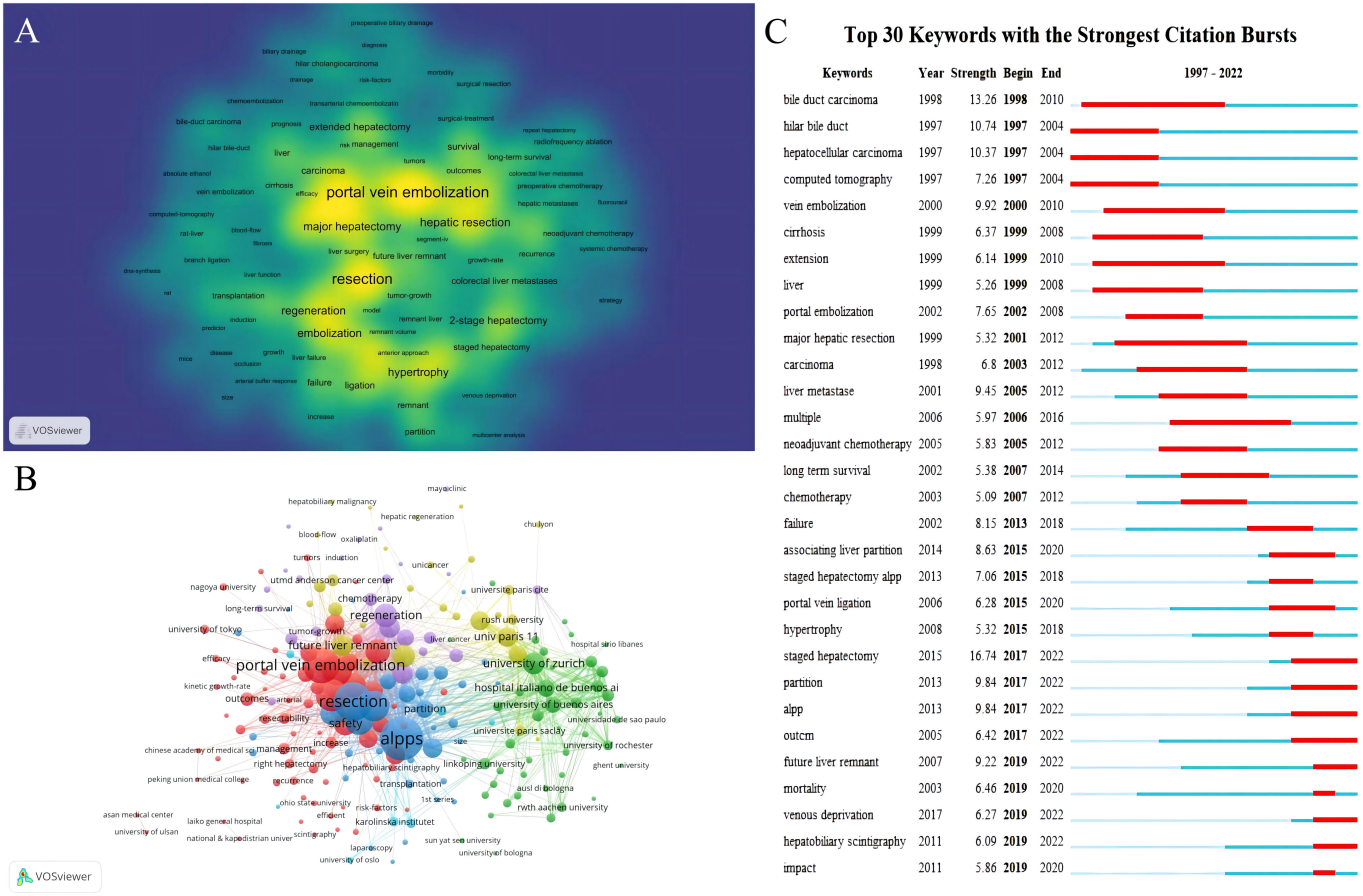
Maps of keywords in the FLR augmentation techniques. **(A)** The density map; **(B)** Co-occurrence network and clusters. **(C)** The burst map. **(A)** Nodes that are darker in color and closer to yellow indicate a higher frequency of the corresponding keyword mentioned. This indicates that more research has been conducted in the related field. **(B)** The size of the circular node in the graph corresponds to the number of publications it represents. The links between the nodes represent the degree of association, and thicker links indicate more frequent cooperation between two nations or organizations. The color of the node corresponds to various clusters. **(C)** The red section represents the time period during which the keyword is mentioned prominently.

We utilized VOSviewer [version 1.6.19] to conduct keyword co-occurrence and network cluster analysis. The software yielded a total of 2069 keywords, from which we selected those with a minimum of 10 occurrences to ensure their significance. Ultimately, we analyzed 155 keywords that met this threshold. These keywords provide insights into the current hotspots in the FLR augmentation technique. Additionally, we identified 5 keywords that appeared over 200 times, with “portal vein embolization” being the most frequent (n=364), followed by “resection” (n=341), “hepatectomy” (n=267), “hepatocellular carcinoma” (n=240), “hepatic resection” (n=205), “major hepatectomy” (n=186), “embolization” (n=183), “hypertrophy” (n=172), “regeneration” (n=170), “cancer” (n=158), and “alpps” (n=154). We also included a density map (**Figure 5A**) that highlights the high-frequency keywords.

Furthermore, **Figure 5B** demonstrated a network cluster analysis outcome based on keywords and institution. Cluster blue and Cluster red mainly contained keywords while Cluster yellow and Cluster green mainly contained institutions. The central location of specific keywords such as portal vein embolization, alpps and safety implies that they have been widely discussed and are among the most significant research directions in the field of preoperative FLR augmentation techniques. Other keywords located on the edge, such as long-term survival, hepatobiliary scintigraphy and kinetic growth rate which may have just emerged in recent years and therefore have less relevant research, can be used as a reference for future development. These institutions which positioned at the center of the cluster, such as University of Zurich, Italian hospital Buenos Aires, and University of Paris 11, indicated their broad involvement in different directions of the field and enjoys a relatively authoritative status in this domain.

To identify any sudden increases in keyword usage, we synthesized all the relevant data from 1997 to 2022 and then extracted 493 samples. Burst keywords, which are keywords that experience a significant surge in usage within a particular period, can be indicative of research hotspots, current and future trends. Thus, this information can not only help us understand the evolution of research hotspots over time but also provide insights into recent and future research trends. **Figure 5C** shows the thirty burst keywords that we discovered.

During the period of 1997-2004, research primarily focused on HCC and hilar cholangiocarcinoma. However, as PVE technique matured and became more commonly used in clinical practice, efforts were made to expand its use to patients with liver cirrhosis or colorectal cancer liver metastasis (23, 29). The effectiveness and safety of combining PVE with neoadjuvant chemotherapy in patients with colorectal cancer liver metastasis requiring extended hepatectomy has become a hot topic of discussion due to the continuous updates of chemotherapy drugs and the increasing use of neoadjuvant therapy as a standard treatment for colorectal cancer in 2005-2010 (30, 31). At the same time, the postoperative complications and long-term prognosis of the FLR augmentation technique have also attracted interest, with key words such as “long term survival” and “failure” appearing repeatedly. Since 2012, ALPPS has become a hot topic of research, with related keywords such as “associated liver partition “, “staged hepatectomy” and “alpps” were repeatedly mentioned as explosive words between 2015 and 2018. After 2019, many new techniques have be used in the clinical practice, with key words such as “Hepatobiliary Scintigraphy” and “Venous deprivation”.

## 4 Discussion

Extended hepatectomy is a widely used curative treatment for various liver diseases, both primary and secondary. The advancement of surgical techniques and perioperative care has made it possible to perform more aggressive resection surgeries. However, the size of the future liver remnant (FLR) is a crucial factor in determining treatment eligibility and postoperative prognosis (32). Regenerative liver surgery aims to increase the size of the FLR before resection surgery to overcome this limitation. This study employs a literature-based method to analyze previously published papers and provide insight into the future direction of preoperative FLR augmentation techniques.

France has made tremendous contributions to preoperative FLR augmentation research. Other notable nations have also done a lot were Japan, the USA and Germany. Despite starting late, Japan has become one of the largest contributors in this field, which may be due to Japan’s high prevalence of viral hepatitis and HCC (33). However, Japan has an average of 28.21 citations per article, which is lower than other European countries. Based on the analysis of institutional collaboration, there is extensive collaboration among international institutions in research on preoperative FLR augmentation techniques, and we consider this to be one of the important factors contributing to the rapid development of this topic.

An analysis of journals and co-cited journals can assist researchers in selecting the most suitable journals for submitting their papers. In the research, HPB emerged as the most frequently published journal (n=65), while Ann Surg was identified as the most commonly co-cited journal (n=8088). Among the top 10 journals, five were located in the Q1 JCR category, and the journal with the highest impact factor (IF) was Ann Surg (IF = 13.787). The findings also showed that of the top 10 co-cited journals, eight were situated in the Q1 JCR division, and the journal with the highest IF was Hepatology (IF=17.298). These results imply that research on the FLR augmentation techniques was favored by many top-quality and high-impact journals, particularly those in the surgical field.

In 1986, Kinoshita et al. introduced the concept of using PVE to induce compensatory liver enlargement and expand the eligibility for resection (3). Although their primary focus was on treating portal tumor thrombi in HCC patients. Shortly thereafter in 1990, Makuuchi et al. presented the initial results of their PVE series, which involved 14 patients with hilar cholangiocarcinoma (4). Their study demonstrated that the technique was safe and feasible in reducing post-resection liver failure. As shown in **Figure 5A**, PVE, the earliest studied preoperative liver augmentation technique, has significantly more related studies than other techniques. Additionally, **Figure 5C** illustrated that between 1998 and 2012, PVE expanded its indications as the technique became increasingly refined. Nevertheless, the slow rate of liver growth is the primary disadvantage of PVE. Some patients may miss the chance for surgery even after undergoing multiple PVE treatments due to tumor progression or failure to achieve sufficient FLR (34, 35).

In 2007, Professor Schlitt unexpectedly performed the first ALPPS procedure in Germany on a patient with hilar cholangiocarcinoma who was initially scheduled for extended right hepatectomy. However, during surgery, it was discovered that the patient’s FLR was too small. Consequently, Schlitt altered the procedure to a left liver duct-jejunum anastomosis and left the liver parenchyma *in situ* by dividing the right edge of the falciform ligament for exposure. He also ligated the right portal vein branch to decrease blood supply to the diseased liver. Eight days after the surgery, CT showed a considerable increase in the volume of the left liver lobe. On the ninth day, the patient underwent extended right hepatectomy successfully. This procedure was subsequently named ALPPS (36). In 2012, Schnitzbauer et al. originally published the comprehensive ALPPS procedure. Subsequent studies have demonstrated that ALPPS produces a greater rate of average FLR hypertrophy and considerably enhances the likelihood of a successful surgery compared to PVE, as evidenced by research findings (6, 16, 17). The rise in the quantity of relevant publications since 2012 depicted in **Figure 1**, as well as “Associating liver partition” became a burst keyword in 2015 according to **Figure 5C**, further confirmed the pioneering significance of the research conducted by Schnitzbauer et al.

However, the drawbacks of ALPPS are significant as well, with high postoperative complication rates and perioperative mortality primarily caused by the presence of two liver transections resulting in bile leakage, abdominal infections, and liver function failure. Research has indicated that the 90-day postoperative mortality rate for bile duct carcinoma after ALPPS is as high as 48%, while the postoperative mortality rate for HCC is around 12% within 90 days. In patients with liver metastasis from colorectal cancer, ALPPS has a postoperative complication rate of about 39% and a 90-day mortality rate of around 7% (37–39).Considering the morbidity and mortality risks associated with the procedure, the use of ALPPS is currently limited to situations where the potential benefits outweigh the potential hazards, as suggested by recent studies (40, 41).

“Venous deprivation” became an burst keyword in 2019, which originated from a case of TIPS performed for refractory ascites presented by Le Roy et al. in 2016 (42). This patient had early complete thrombosis in the right hepatic vein and right portal vein, progressive and complete atrophy of the right liver, and marked hypertrophy of the left liver due to the obstruction by TIPS. Although liver function was initially poor, it gradually improved until complete recovery. This finding brings a new idea to the regenerative liver surgery, as the combination of PVE and hepatic vein embolization (HVE) to achieve a rapid increase in FLR. In the same year, Guiu et al. first completed seven cases of PVE combined with HVE and officially named it as Liver venous deprivation (LVD) (6). A follow-up research was conducted on 12 patients with Klatskin tumor to compare PVE and LVD. Results showed that LVD had a significantly higher FLR hypertrophy rate (58%) compared to PVE (37%) using a standardized FLR ratio. Furthermore, there was a tendency towards shorter postoperative hospital stay and 90-day mortality, although not statistically significant (43). Panaro et al. found that the rapid increase in liver volume in LVD was accompanied by a gradual increase in liver function, with no non-growth or delayed growth occurring. At the same time, liver function indexes, especially transaminases, bilirubin and prothrombin time, recovered extremely fast, and there was no immature liver proliferation in ALPPS (44).

To summarize, PVE continues to be a well-established and highly efficient method for inducing liver hypertrophy before surgical intervention in patients diagnosed with a range of hepatobiliary malignancies such as HCC, Cholangiocarcinoma, and Colorectal liver metastasis. According to recent systematic reviews and meta-analyses, the reported mean rates of FLR hypertrophy range from 37.9% to 49.4%, with the success rates of hepatectomy ranging between 75.9% and 96.1%. The incidence of major complications in cases was found to be between 2.2% and 3.1%, and the mortality rate was less than 0.1%, according to these studies (45–47). PVE may not be effective in all cases, and there is a significant concern about the potential for the stimulation of tumor growth after the procedure. This issue remains poorly understood but could have catastrophic consequences (34, 35).

ALPPS was regarded as a significant advancement in regenerative liver surgery. In 2018, The LIGRO study compared ALPPS to two-stage hepatectomy (TSH) with PVE/PVL (17). The study revealed that the resection rate increased after ALPPS compared to conventional methods (92% in ALPPS vs. 57% in TSH, p < 0.001). A subsequent assessment also indicated the oncological superiority of ALPPS, as the higher transection rate in ALPPS was directly associated with significantly better median survival (46 months in ALPPS vs. 26 months in TSH, p = 0.028). Recent studies have reported that the mean FLR hypertrophy rates of ALPPS range from 63.0% to 87.2%, and the rates of successful hepatectomy were between 92.0% and 100.0%. However, major complications have been observed in 40.0% to 71.4% of cases, with mortality rates ranging from 9.0% to 28.7% (16, 17, 48–50). Despite the benefits of rapid hypertrophy to improve resectability and survival in metastatic liver tumors, ALPPS is not versatile enough due to its low safety profile. Therefore, it is not a suitable option for an aging patient population or for primary liver tumors such as HCC and cholangiocarcinoma. However, ALPPS has played a significant role in developing the concept of rapid hypertrophy.

LVD, as an emerging planned hepatectomy for liver augmentation, the results of several studies have suggested that it is less invasive, safer, can rapidly promote FLR proliferation, meet the criteria for phase II surgery in a shorter waiting period. LVD was suitable for a range of hepatobiliary malignancies just like PVE, but the FLR hypertrophy rates and successful hepatectomy rates were superior to PVE. At the same time, LVD has a much lower major complications and mortality rates than ALPPS. It may become a hot research topic in the field of liver tumor treatment.

Moreover, new techniques will likely become prominent areas of research in the future. For example, Hepatobiliary Scintigraphy has the potential to enhance the precision when evaluating FLR increase post-surgery (51) and while Radiation Lobectomy can be used as a locoregional treatment for hepatic malignancy while simultaneously inducing FLR hypertrophy (52).

In terms of limitations, our analysis focused only on English articles published in the WoSCC. This approach may have resulted in the exclusion of significant publications. Nevertheless, the WoSCC is a widely utilized database for bibliometric analysis that encompasses a vast amount of data. Another limitation is that recently published articles of high quality may not have had sufficient time to accumulate citations and, therefore, were not included in our analysis.

## 5 Conclusions

The objective of this investigation is to present a bibliometric analysis that provides an outline of the advancements in FLR augmentation technique research publications. Our analysis highlighted the most notable studies, countries, institutions, journals, and authors to indicate the most influential research in this field. We anticipate that our findings will be beneficial in determining the future direction of studies related to FLR augmentation technique. Furthermore, there is a need for further technical advancements to enhance safety and efficacy, as well as more clinical trials to validate these findings.

## Data availability statement

The original contributions presented in the study are included in the article/supplementary material. Further inquiries can be directed to the corresponding author.

## Author contributions

SD: data collection and drafting of the manuscript. SD: drafting and revision. ZW: data collection. ZW and DL: design of this work and data analysis. All authors contributed to the article and approved the submitted version.
